# Comparative Transcriptome Analysis Revealed the Freezing Tolerance Signaling Events in Winter Rapeseed (*Brassica rapa* L.)

**DOI:** 10.3389/fgene.2022.871825

**Published:** 2022-04-26

**Authors:** Wangze Wu, Haobo Yang, Peng Xing, Yun Dong, Juan Shen, Guofan Wu, Sheng Zheng, Lingling Da, Jiangtao He, Yujun Wu

**Affiliations:** ^1^ College of Life Sciences, Northwest Normal University, Lanzhou, China; ^2^ Crop Research Institute, Gansu Academy of Agriculture Sciences, Lanzhou, China; ^3^ Ministry of Education Key Laboratory of Cell Activities and Stress Adaptations, School of Life Sciences, Lanzhou University, Lanzhou, China

**Keywords:** *Brassica rapa* L., cell membrane, freezing stress, MAPK signaling, transcriptome

## Abstract

Winter rapeseed (*Brassica rapa* L.) is an important oilseed crop in northwest China. Freezing stress severely limits its production and geographical distribution, and frequent extreme freezing events caused by climate change are increasing the chances of winter freeze-injury. However, the underlying mechanism of *B. rapa* response to freezing stress remains elusive. Here, *B. rapa* genome (v3.0) was used as a reference for the comparative transcriptomic analysis of Longyou 6 and Tianyou 2 (strong and weak cold tolerance, respectively) under different freezing stress. Before and after freezing stress, 5,982 and 11,630 unique differentially expressed genes (DEGs) between two cultivars were identified, respectively. After freezing stress, the GO terms in Tianyou 2 were mainly involved in “macromolecule biosynthetic process”, and those in Longyou 6 were involved in “response to stimulus” and “oxidoreductase activity”. Morphological and physiological results indicated that Longyou 6 retained a higher basal freezing resistance than Tinayou 2, and that cold acclimation could strengthen the basal freezing resistance. Freezing stress could activate the MAPK signal cascades, and the phosphorylation level of Longyou 6 showed a higher increase in response to freezing treatment than Tianyou 2. Based on our findings, it was speculated that the cell membrane of *B. rapa* perceives external signals under freezing stress, which are then transmitted to the nucleus through the cold-activated MAPK cascades and Ca^2+^-related protein kinase pathway, thus leading to activation of downstream target genes to enhance the freezing resistance of *B. rapa.*

## Introduction

Low temperature is an important environmental factor that negatively affects plant growth, development, crop productivity, quality, and geographic distribution (Thomashow, 1999). However, plants have evolved sophisticated regulatory mechanisms that allow them to adapt to unfavorable environments through a series of physiological and molecular changes (Hare et al., 1998; Shi et al., 2018). In nature, plants need to withstand extended periods of exposure to low temperatures in late autumn and early winter to acquire increased freezing tolerance, the process of which is called cold acclimation. In contrast, when exposed to warmer temperatures in spring, the loss of cold tolerance is called cold deacclimation (Thomashow, 1999), which makes plants more susceptible to freezing stress induced by frost events in early spring (Vitra et al., 2017). Therefore, understanding the molecular mechanisms of how plants adapt to sudden extreme freezing events will provide valuable information and genetics resources for improving low temperature tolerance in crops.

Cold stress includes chilling stress (0–15°C) and freezing stress (<0°C) (Thomashow, 1999). Maize (*Zea mays*), rice (*Oryza sativa*), and potato (*Solanum tuberosum*) originate from tropical and subtropical regions and are chilling-sensitive, whereas plants that originate from temperate climates, such as wheat (*Triticum aestivum*), rye (*Secale cereale*), and barley (*Hordeum vulgare*), have evolved sophisticated cold acclimation mechanisms to adapt to low temperatures and survive freezing temperatures (Chinnusamy et al., 2007). During the cold acclimation process, a series of comprehensive physiological and biochemical events occur. Among them, regulating the expression of cold stress-related genes and transcription factors are critical for plants to cope with low temperatures stress (Hincha and Zuther, 2020).

Over the past two decades, numerous transcription factors have been identified in cold stress signal pathways in model plants *Arabidopsis* and rice (Ding et al., 2019). Those transcription factors mainly include AP2/ERF, bHLH, ZFP, MYB, WRKY, and NAC. Among them, the C-repeat binding factor (CBF)-dependent cold signaling pathway is the best-characterized transcription factor. CBF/DREB1 (dehydration responsive element binding factor 1) belongs to the AP2/ERF transcription family, which CBFs proteins can bind to the C-repeat/dehydration response element (CRT/DRE) of cold-regulated (COR) genes and regulate their expression (Liu et al., 1998; Chinnusamy et al., 2003). Whereas the expression of CBFs is also regulated by other transcription factors. For example, the inducer of CBF expression 1 (ICE1) is an MYC-type bHLH transcription factor family protein that is the best-characterized transcriptional activator of *CBFs* genes to date, it can positively regulate *CBFs* expression and freezing tolerance by directly binding to the *CBF3* promoter region (Chinnusamy et al., 2003). Moreover, calmodulin-binding transcription activator (CAMTA) proteins (CAMTA1-5) can positively regulate *CBF1* and *CBF2* expression (Kidokoro et al., 2017). Interestingly, some transcription factors, such as MYB15, which is a member of the R2R3 subfamily, can also negatively regulate the expression of *CBFs* by directly binding to the conserved MYB motif in their promoter to respond to cold stress (Agarwal et al., 2006). Besides transcriptional regulation, post-transcriptional and post-translational regulation are vital in the *CORs* genes expression regulatory mechanism (Ding et al., 2019).

Recently, cold stress response mechanisms of different crops at the transcriptional level have been clarified. Up to now, the differentially expressed genes (DEGs) of various plants under cold stress, such as *Arabidopsis*, rice, maize, and wheat, have been studied at the whole-genome level (Kreps et al., 2002; Winfield et al., 2010; Shen et al., 2014; Mao et al., 2017). The freezing regulation mechanism of plants is a complex quantitative trait, with different species possibly exhibiting different cold response mechanisms (Ding et al., 2019). For instance, in *Arabidopsis*, cold-activated MPK3 and MPK6 phosphorylate ICE1 negatively regulates the cold tolerance. However, in rice, cold-activated OsMAPK3 phosphorylates OsbHLH002/OsICE1 positively regulates the chilling tolerance. Moreover, overexpression of tomato *LeCBF1* in *Arabidopsis* increases the freezing tolerance of *Arabidopsis*, but overexpression of *LeCBF1* or *AtCBF3* in tomato does not increase its freezing tolerance (Zhang et al., 2004). Over the past several decades, CBF-dependent cold signaling pathways have been extensively studied in the model species *Arabidopsis* and rice, but neither *Arabidopsis* nor rice can adapt to a continuous life cycle in extreme winter climates.

Winter rapeseed (*Brassica rapa* L.) is an important cruciferous oilseed crop in northwest China. *Brassica* species are closely related to the model plant *Arabidopsis* (Yang et al., 1999) and possess a smaller genome (Wang et al., 2011; Zhang et al., 2018) than that of *Brassica napus* L. (Chalhoub et al., 2014). Moreover, *B. rapa* is an overwintering herbaceous plant, notably, some *B. rapa* varieties can survive at extremely low temperatures (−32°C, overwinter survival rate of 80–95%), therefore, *B. rapa* might be a “perfect” model crop for uncovering cold signaling in crops. Unfortunately, the molecular mechanisms by which *B. rapa* adapts to freezing stress remain poorly understood. Several studies have conducted transcriptome analysis of *B. rapa* under cold stress using a low accuracy *B. rapa* genome v1.5, v2.0, or v2.5 as the reference genome based on 4°C cold stress transcriptome analysis (Ma et al., 2019; Fang et al., 2021). Recently, cold-responsive microRNA and metabolic change under freezing stress (-4°C, 8 h) have been reported as possible mechanisms of *B. rapa* response to freezing stress (Xu et al., 2018; Zeng et al., 2018). However, the cold resistance of crops is a complex qualitative trait, and these varieties have a complex genetic background, therefore the underlying mechanism of *B. rapa* response to freezing stress remains elusive. In nature, sudden sub-zero temperature conditions, such as frost, cause the most serious freezing injury to *B. rapa*. To investigate the molecular mechanism of *B. rapa* under sub-zero freezing stress, we analyzed and compared the transcriptomes of Longyou 6 (strong cold tolerance) and Tianyou 2 (weak cold tolerance) under short periods of sub-zero freezing stress using the newly assembled, high accuracy *B. rapa* genome v3.0 as the reference genome, which is based on single-molecule sequencing technology (Zhang et al., 2018). This study will be a valuable resource for understanding the freezing stress signaling pathway of *B. rapa* and for breeding cold-tolerant *B. rapa* varieties.

## Materials and Methods

### Plant Materials and Growth Conditions

The *B. rapa* varieties Longyou 6 (strong cold tolerance), Tianyou 2 (weak cold tolerance), and Westar were obtained from Gansu Agriculture University and Gansu Academy of Agriculture Sciences. *B. rapa* seeds were maintained at 4°C in ddH_2_O for 3 days for low-temperature vernalization, and the seeds were then grown on soil in a growth chamber at 22°C for 14 days under a 16 h light/8 h dark photoperiod. For cold-acclimated (CA) treatment, 14-day-old seedlings were treated at 4°C for 72 h. The seedings of CA and NA (non-acclimated) were transferred to a freezing chamber for freezing assays at 0, −4, and −8°C for 3 h, respectively. The freezing program began at 0°C, with the temperature decreasing by 1°C per hour until the temperatures described above were reached. After freezing treatment, the seedlings were shifted to 4°C and kept in darkness for 12 h and then transferred to normal conditions (16 h light/8 h dark) at 22°C for another 72 h of recovery. The seedlings’ phenotype at each freezing temperature were photographed, and the survival rates (Ye et al., 2019), ion leakage (Shi et al., 2012), malondialdehyde (MDA) (Dhindsa et al., 1981), proline contents (Bates et al., 1973), soluble proteins (SP) contents (Bradford, 1976), peroxidase (POD) (Conklin and Smith, 1971) and superoxide dismutase (SOD) activities (Weydert and Cullen, 2010) were measured. For transcriptome and qRT-PCR analyses, the leaves and stems of 14-day-old Longyou 6 and Tianyou 2 seedlings grown at 22°C were collected as the controls CKL and CKT, respectively. The 14-day-old NA and CA seedlings of Longyou 6 and Tianyou 2 were freezing treated at 0 °C, −4°C, and −8°C for 3°h, respectively. Then the seedlings of each cultivar at different freezing treatment were collected to mix as SLD and STD, respectively. These materials were flash-frozen in liquid nitrogen and stored at −80°C for transcriptome sequencing and qRT-PCR analysis.

### Total RNA Extraction and Transcriptome Sequencing

The samples were sent to the BGI (Shenzhen, China) Biological Company for RNA isolation, detection, and sequencing. Illumina HiSeq was used for transcriptome sequencing.

### Transcriptome Assembly and Annotation

This study used SOAPnuke (v1.4.0) software to count the original sequencing data and Trimmomatic (v 0.36) software to filter the data. The specific steps were as follows: (1) remove reads containing linkers (linker contamination); (2) remove reads with an unknown base N content greater than 5%, and (3) remove low-quality reads (the proportion of bases with a quality value of less than 15 in a particular read was greater than 20%).

*B. rapa* genome v3.0 (http://brassicadb.cn/#/Download/) was used as the reference for subsequent transcriptome data analysis. Bowtie 2 (v2.2.5) software was used to align clean reads to the reference gene sequence, and RSEM was used to calculate the expression levels of genes and transcripts. After obtaining the clean reads, we used Hierarchical Indexing for Spliced Alignment of Transcripts (HISAT; v2.1.0) to align the clean reads to the reference genome sequence. StringTie (v1.0.4) was used to reconstruct the transcript of each sample, and Cufflinks (v2.2.1) was used to compare the integrated transcript with the reference annotation information and define the transcript with the following characteristics as a new transcript: (1) unknown but located in intergenic regions; (2) located in the intron regions of known genes; (3) transcripts that have a particular intersection with the exons of known genes; and (4) potential new transcripts or transcription fragments (with at least one junction; the locus is consistent with the reference gene).

### Annotation of Transcription Factor Genes

Getorf (EMBOSS:6.5.7.0) was used to detect the open reading frame (ORF) of Unigene, and Hmmsearch (v3.0) was used to align the ORF with the transcription factor protein domain, which was identified based on characteristics of the transcription factor family described by PlantTFDB (http://planttfdb.gao-lab.org/index.php).

### Identification of DEGs

DEGs detection was performed according to the method described by Wang et al. (2009). To improve the accuracy of DEGs, we identified genes with expression level differences greater than two times and a Q-value of ≤ 0.001 and screened them as significant DEGs.

### GO and KEGG Enrichment Analyses

Based on the annotation results of GO and KEGG and the official classification, we classified DEGs into functions and biological pathways and used the phyper function in R software for the enrichment analysis of GO and KEGG pathways, respectively. The p-value was corrected, and the functions with Q values ≤ 0.05 were interpreted as indicative of significant enrichment.

### Gene Expression Verification

To confirm the validity of the RNA sequencing data, we randomly selected 16 cold-response DEGs and 20 differentially expressed transcription factors for qRT-PCR analysis. Total RNA was extracted from the stems and leaves using the TRIzol method, and cDNA was synthesized using a reverse transcription kit (TaKaRa, Japan). Primer3 Plus (http://www.bioinformatics.nl/cgi-bin/primer3plus/primer3plus.cgi) was used to design gene-specific primers, which are listed in Supplementary Table S3. The relative change in expression was calculated using 2^−ΔCt^, and BraActin (BraA03g004000.3C) was used as an internal reference gene, with three biological replicates were used for each sample.

### Protein Extraction and Immunoblotting

*B. rapa* seedlings were grown on 1/2 MS medium, containing 0.8% agar, at 22°C (16 h light/8 h) for 14 days and exposed to the freezing treatment. The freezing treatment protocol is similar to those in the plant materials and growth conditions section mentioned above. Materials were collected from the NA treatment and CA treatment groups (pretreated at 4°C for 72 h) at −4°C (3 h) and −8°C (3 h) to extract total proteins with a protein extraction buffer (50 mM Tris–HCl pH 7.5, 150 mM NaCl, 1% Triton X-100, 1 mM PMSF, 1 mM EDTA, 20 mM NaF, and 1× protease inhibitor cocktail). The protein concentration of each sample was adjusted to the same level using Quick Start Bradford Dye Reagent (Bio-Rad, California, United States). Total proteins were separated via 10% SDS/PAGE and transferred onto PVDF membranes (Bio-Rad). Immunoblotting was performed using anti–Phospho-p44/p42 MAPK (anti-pTEpY) (1:2,000, Cell Signaling Technology, Danvers, United States).

## Results

### Phenotypic and Physiological Responses to Freezing Stress

**FIGURE 1:**
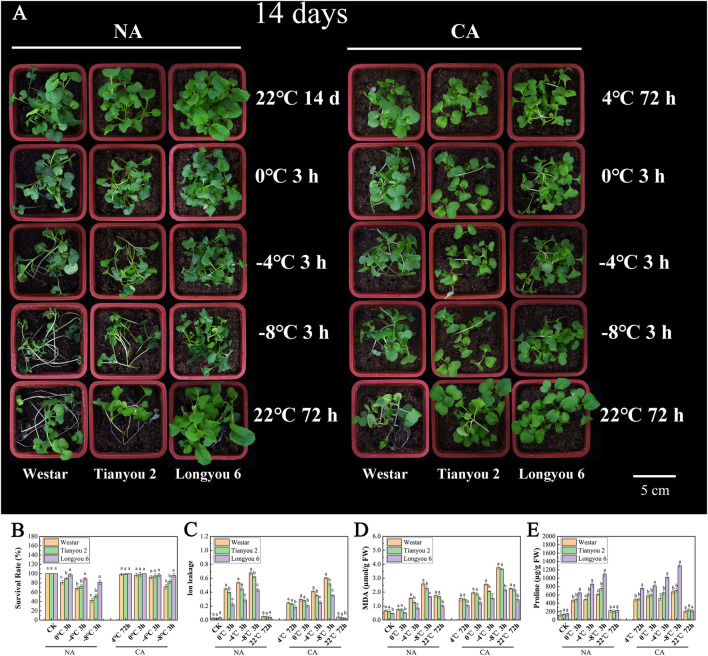
The morphological and physiochemical characterization of *B. rapa* under different freezing treatments. **(A)** The morphological characterization of *B. rapa* under different freezing treatments. **(B–E)** The physiochemical indicators of *B. rapa* under different freezing treatments. Non-acclimated (NA) freezing treatment seedlings **(A)** left, Cold-acclimated (CA) freezing treatment seedlings **(A)** right. Data were means of three in dependent biological replicates (*n* ≥ 20 for each repeat) ± SE. Lowercase letters present significant difference at *p* < 0.05 (one-way ANOVA).

To evaluate the resistance of Longyou 6 and Tianyou 2 to freezing stress, the phenotypic and physiological indicators of 14-day-old non-acclimated (NA) and cold-acclimated (CA) seedlings were examined under different freezing treatments. The results showed that the phenotypes of Longyou 6 and Tianyou 2 did not differ significantly from those of the control (Westar) at 22°C (Figure 1A). However, in the NA treatment after 3 h at 0°C chilling, the leaves of Tianyou 2 and Westar began to wilt, and lodging occurred in some plants; after 3 h of freezing treatment at −4°C, the symptoms of freezing injury intensified in Tianyou 2 and Westar. The symptoms of freezing damage were more severe in Tianyou 2 and Westar after the −8°C freezing treatment, with the seedlings being completely lodged and leaves severely wilted. However, in the NA treatment, the 14-day-old seedlings of Longyou 6 did not exhibit freezing damage symptoms until after 3 h at −8°C freezing treatment (Figure 1A; Supplementary Figure S1). Intriguingly, after 24 h of 4°C CA treatment, Longyou 6 and Tianyou 2 did not show apparent freezing injury symptoms after 3 h at 0°C or −4°C freezing treatment, but Westar showed mild freezing injury symptoms after 3 h at −4°C. After 3 h at −8°C, Tianyou 2 and Westar exhibited apparent freezing injury symptoms, while Longyou 6 showed slight freezing injury symptoms. According to survival rate statistics, Longyou 6 exhibited a significantly greater basal freezing tolerance than Tianyou 2 and Westar under both NA and CA conditions, and freezing tolerance was enhanced by CA treatment (Figure 1B). CA treatment also improved the freezing tolerance of Tianyou 2 and Westar, but to a lower degree than in Longyou 6, at least under our experimental conditions.

As a series of comprehensive physiological and biochemical events occur under cold stress, we examined the changes in physiological indicators of NA and CA seedlings under freezing treatments. Our data showed that the ion leakage from Westar and Tianyou 2 was much higher than that from Longyou 6 under both NA and CA treatments (Figure 1C), indicating that the plasma membrane damage caused by freezing stress was more severe in Westar and Tianyou 2. Moreover, the content of MDA, an important indicator of membrane injury, was significantly lower in Longyou 6 than in Westar and Tianyou 2 (Figure 1D). Similarly, the proline, SP content, POD and SOD activities of Longyou 6 were much higher than that of Tianyou 2 and Westar, both with and without cold acclimation (Figure 1E and Supplementary Figure S2). These results indicated that the Westar and Tianyou 2 seedlings had severe membrane damage after freezing stress. As expected, after CA treatment, the plants exhibited lower ion leakage and higher protective substance content than those after NA treatment; however, the trend was more obvious in Longyou 6. These phenotypic observations suggest that Longyou 6 may have a stronger basal freezing resistance than Tinayou 2, and cold acclimation can confer Longyou 6 with strong freezing resistance.

### Overview of Transcriptome Data

**FIGURE 2:**
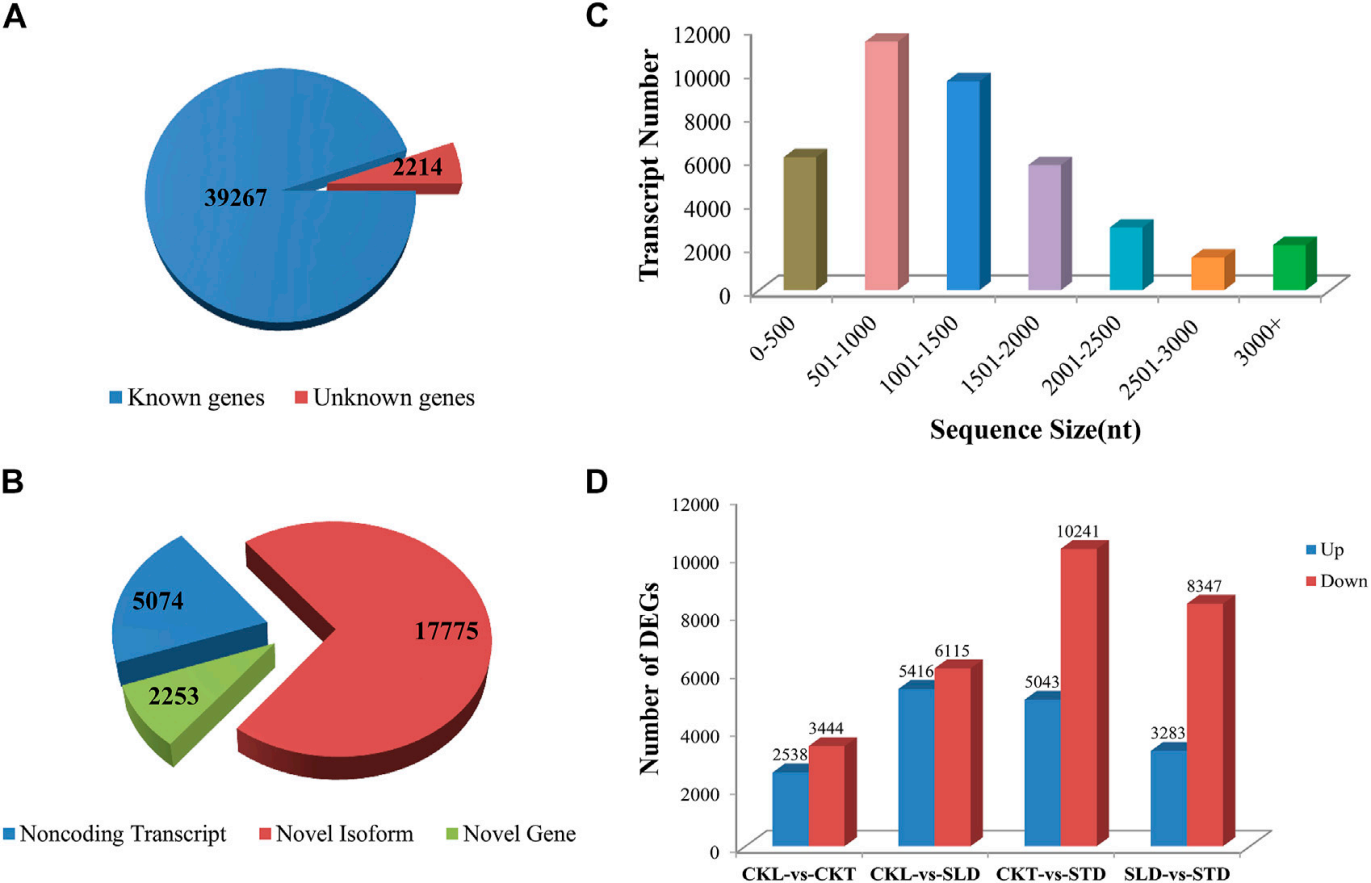
Overview of transcriptome data in *B. rapa* under freezing treatments. **(A)** The total number of detected expressed genes. **(B)** The number of new transcripts detected. **(C)** The length of the detected transcripts. **(D)** The number of DEGs in CKL *vs*. CKT, CKL *vs*. SLD, CKT *vs*. STD, and SLD *vs*. STD.

An *Arabidopsis* cold-responsive transcriptome study shown that 3.9% of *Arabidopsis* genes are regulated by cold stress. Among these, 74% of cold-regulated genes were regulated by 24 h of cold treatment at 0°C; however, only 26% were regulated by 3 and 6 h of cold treatment at 0°C (Lee et al., 2005). Thus, these results suggest that most of the cold-upregulated genes were late-response genes. Under natural conditions, plants undergoing cold acclimation are frequently exposed to sub-zero temperatures during the autumn, winter, and spring seasons. Therefore, to obtain the actual pattern of natural gene expression data of *B. rapa* under freezing stress, the seedlings at different freezing temperature time points were pooled for each cultivar for transcriptome sequencing. In this study, 12 samples were analyzed using the Illumina HiSeq platform. After removing low-quality reads, each sample produced an average of 11.05 Gb of data. The GC content of each treatment was approximately 48%, the Q30 values were all above 86%, and approximately 83% of clean reads mapped to the reference genome (Supplementary Tables S1, S2). The total number of detected expressed genes was 41,481, of which 39,267 were known genes and 2,214 were predicted new genes (Figure 2A); 25,102 new transcripts were detected, of which 17,775 belonged to new alternative splicing subtypes of known protein-coding genes, 2,253 belonged to new protein-coding gene transcripts, and the remaining 5,074 belonged to long non-coding RNA (lncRNA) (Figure 2B). The detected transcripts were generally of 0–2000 nt (Figure 2C).

Analysis of the DEGs showed that the expression levels of genes in the two varieties changed significantly before and after freezing treatment. Tianyou 2 (control, CKT vs. freezing treatment, STD) had 15,284 DEGs after freezing treatment, among which the numbers of upregulated and downregulated genes were 5,043 and 10,241, respectively. Although Longyou 6 (control, CKL vs. freezing treatment, SLD) had more DEGs (11,531) after the freezing treatment, the number of upregulated and downregulated genes was similar, at 5,416 and 6,115, respectively. Concurrently, a comparison of the two varieties revealed that before the freezing treatment, the number of DEGs between the two varieties was 5,982 (CKL *vs*. CKT), and after the freezing treatment, it reached an astonishing 11,630 (SLD *vs*. STD) (Figure 2D and Supplementary Figure S3).

Statistical analysis was conducted on the reference genome for single nucleotide polymorphisms, alternative splicing, and InDel sites in each sample. The single nucleotide polymorphisms (SNP) mainly included the substitution of A-G and C-T and were concentrated in the exon region, which is also the central location for InDel sites. In addition, five main types of alternative splicing were found in all samples, and the three types known as skipped exon (SE), alternative 3′ splicing site (A3SS), and retained intron (RI) had the most significant number (Supplementary Figure S3).

### Gene Ontology Enrichment Analysis of DEGs

GO annotation analysis was performed on the DEGs detected before and after the freezing treatment of the two varieties. According to the revised *p* value, 24,502 and 35,507 DEGs in Longyou 6 (CKL vs. SLD) and Tianyou 2 (CKT vs. STD) were divided into 35 GO terms (Supplementary Figure S4). DEGs between the two varieties were then analyzed. Before the freezing treatment, 11,409 DEGs between the Longyou 6 and Tianyou 2 varieties (CKL vs. CKT) were divided into 33 GO terms. After freezing treatment, 24,892 DEGs between the two varieties (SLD vs. STD) were divided into 36 GO terms. These GO terms could be further divided into three functional categories: molecular function, cellular component, and biological process. In Longyou 6 (CKL vs. SLD) and Tianyou 2 (CKT vs. STD), 6,869 and 9,693 DEGs were classified into molecular function; 9,771 and 14,569 DEGs belonged to cellular component; and the remaining 7,862 and 11,245 belonged to biological process. A statistical analysis of the GO terms between the two varieties revealed that before the freezing treatment, there were 3,311; 4,315; and 3,783 DEGs between the two varieties (CKL *vs*. CKT) that belonged to molecular functions, cellular components, and biological processes, respectively. After freezing treatment (SLD *vs*. STD), these values were 7,269; 9,613; and 8,010.

**FIGURE 3:**
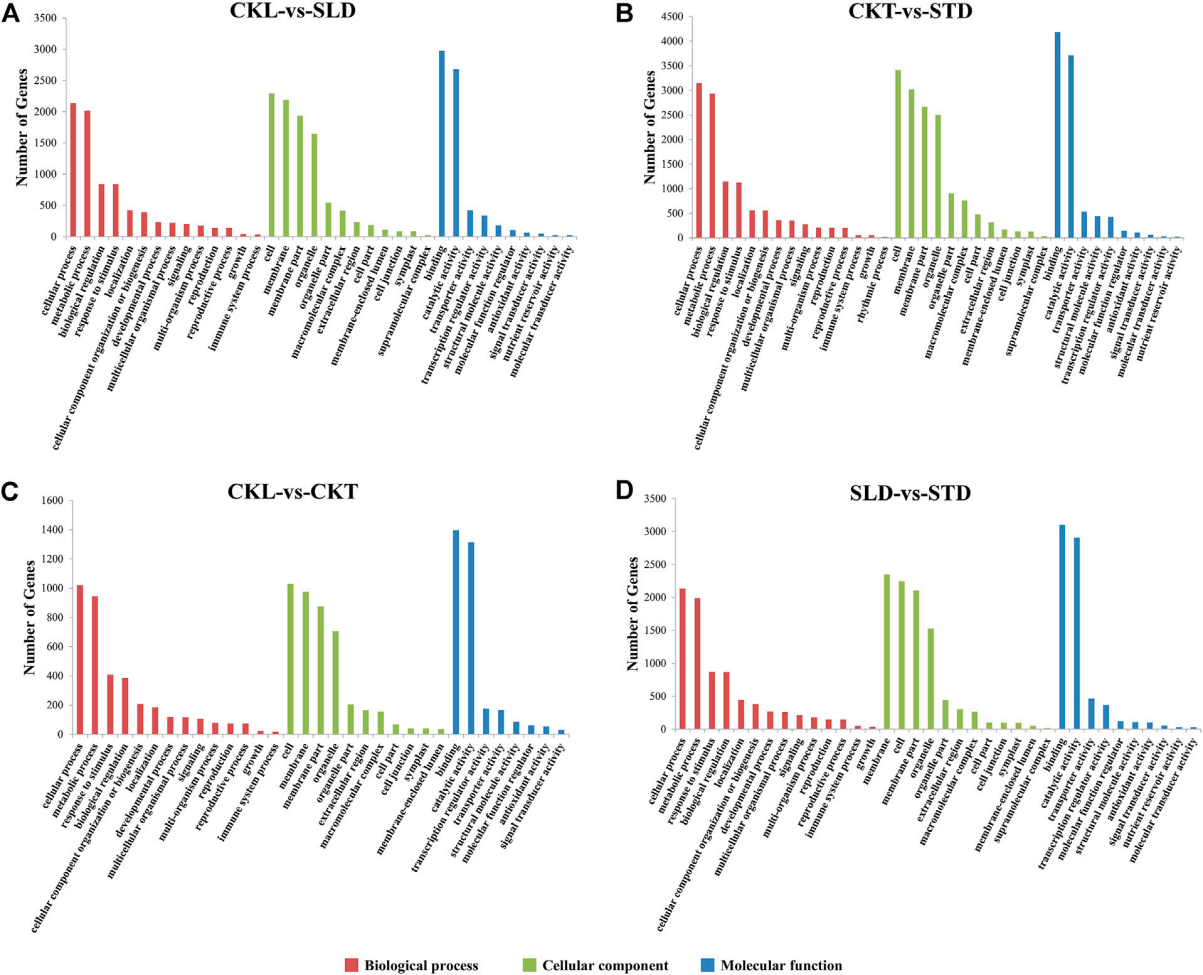
GO enrichment analysis of DEGs in *B. rapa* under freezing treatments. The GO terms in CKL *vs*. SLD **(A)**, CKT *vs*. STD **(B)**, CKL *vs*. CKT **(C)**, and SLD *vs*. STD **(D)**. It is divided into 3 categories: Biological process, Cellular component and Molecular function which are represented by red, light green, and blue respectively.

Based on these results, we further verified GO terms with a high degree of integration (DEGs greater than 10) (Figure 3). Before and after the freezing treatment, the GO terms of the two varieties were relatively similar. The main GO terms in the molecular function category were “binding” and “catalytic activity; ” those in the cell component category were “cell,” “membrane,” “membrane part,” and “organelle; ” and those in biological processes were “cellular process” and “metabolic process.” In addition, according to the bubble chart, after freezing treatment, the most significantly enriched GO terms in Longyou 6 (CKL vs. SLD) were “response to stimulus” (GO:0050896) and “oxidoreductase activity” (GO:0016491) (Figure 3A and Supplementary Figures S5, S6). In contrast, the GO terms that were the most significantly enriched in Tianyou 2 (CKT vs. STD) were “macromolecule biosynthetic process” (GO:0009059), “cellular macromolecule biosynthetic process” (GO:0034645), and “cellular nitrogen compound biosynthetic process” (GO:0044271) (Figure 3B). Between the two varieties, the GO term that was the most significantly enriched before freezing treatment (CKL vs. CKT) was “oxidoreductase activity” (Figure 3C). After freezing treatment (SLD vs. STD), it was “intrinsic component of membrane” (GO:0031224) or “integral component of membrane” (GO:0016021) (Figure 3D and Supplementary Figure S6).

### KEGG Pathway Enrichment Analysis of DEGs

To understand the functions of the DEGs, we mapped the DEGs to reference canonical pathways in the KEGG database. The KEGG metabolic pathway involved in genes was divided into five major branches: “cellular processes”, “environmental information processing”, “genetic information processing”, “metabolism”, and “organic systems”, and further statistically classified under each branch. After freezing treatment, the DEGs in Longyou 6 (CKL *vs*. SLD) and Tianyou 2 (CKT vs. STD) were mainly allocated to 19 KEGG pathways. Most of the pathways were classified as “metabolism”, which included “carbohydrate metabolism”, “biosynthesis of secondary metabolites”, “amino acid metabolism”, and “lipid metabolism”, and the remaining were classified as “genetic information processing” and “environmental information processing”. The statistical results of the KEGG channel between the two varieties (CKL *vs*. CKT and SLD *vs*. STD) before and after the freezing treatment were consistent with the above results (Supplementary Figure S7).

**FIGURE 4:**
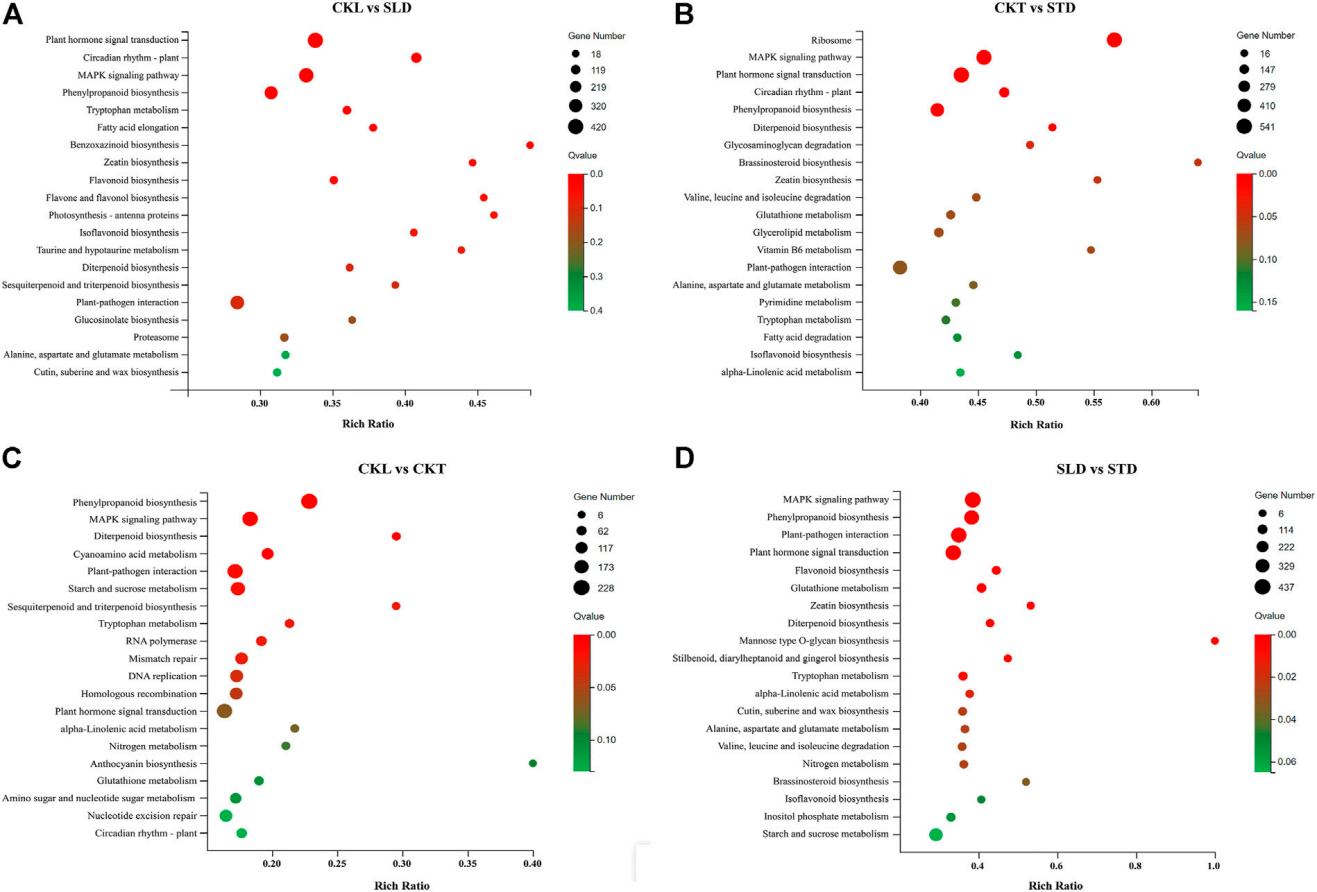
KEGG pathway enrichment analysis of DEGs in *B. rapa* under freezing treatments. The KEGG metabolic pathway in CKL *vs*. SLD **(A)**, CKT *vs*. STD **(B)**, CKL *vs*. CKT **(C)**, and SLD *vs*. STD **(D)**.

According to the results of the bubble chart, after freezing treatment, the most significant enrichment pathways for Longyou 6 (CKL *vs*. SLD) included “plant hormone signal transduction” (Ko04075), “MAPK signaling pathway-plant” (Ko04016), “plant–pathogen interaction” (Ko04626), and “phenylpropanoid biosynthesis” (Ko00940), and the most significant enrichment pathways in Tianyou 2 (CKT *vs*. STD) were “plant hormone signal transduction”, “ribosome “(Ko03010), and “MAPK signaling pathway-plant” (Figures 4A,B). Before the freezing treatment, the most significant enrichment pathways between the two varieties (CKL *vs*. CKT) were “phenylpropanoid biosynthesis”, “MAPK signaling pathway-plant”, “plant–pathogen interaction”, and “plant hormone signal transduction” (Figure 4C). After freezing treatment (SLD vs. STD), the most significant enrichment pathways were “MAPK signaling pathway-plant”, “plant hormone signal transduction”, “plant–pathogen interaction”, and “phenylpropanoid biosynthesis” (Figure 4D).

### Differentially Expressed Transcription Factors Involved in Freezing Stress Response

**FIGURE 5:**
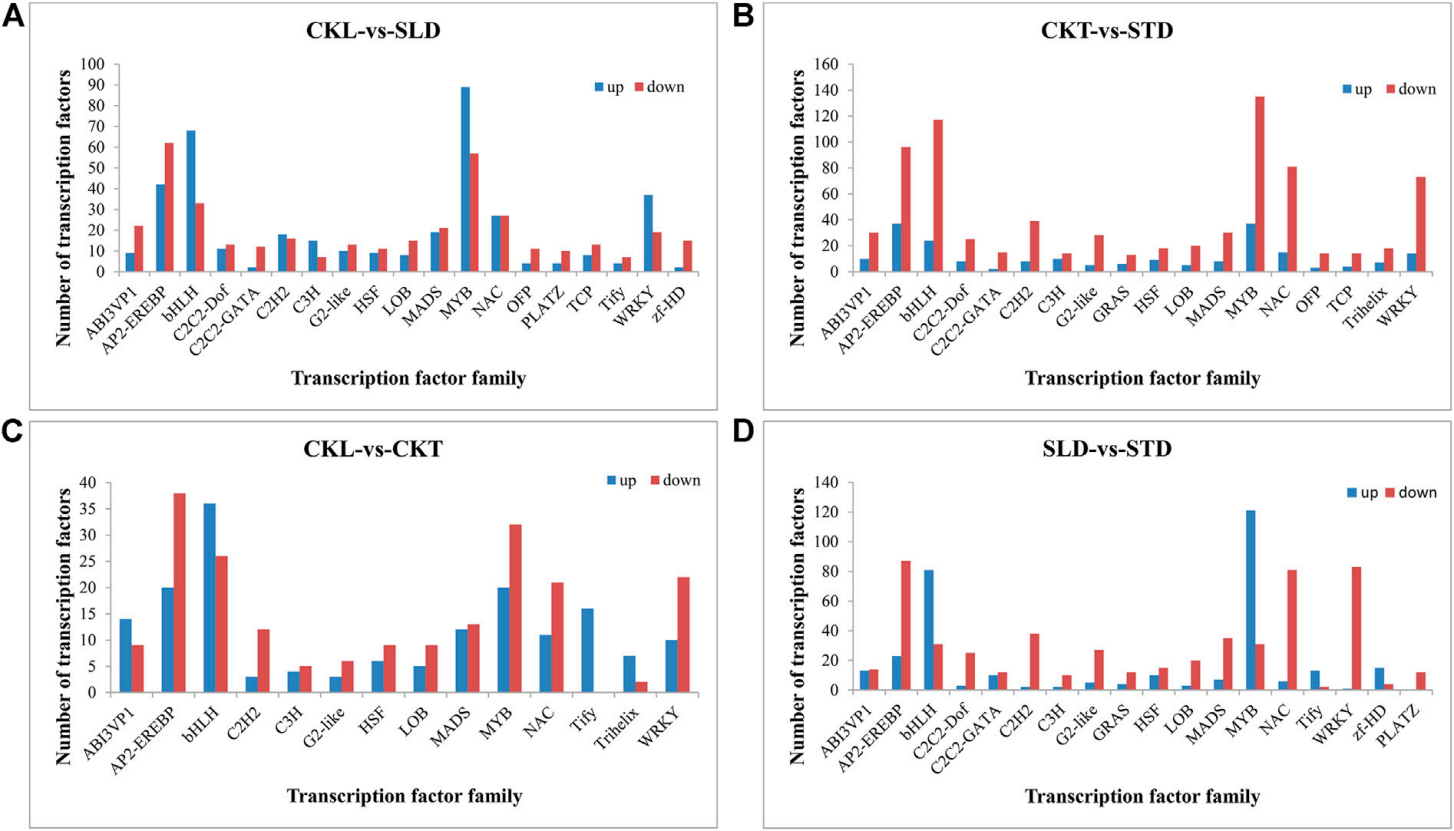
Comparison of major differentially expressed transcription factor families in *B. rapa* under freezing treatments. The differentially expressed transcription factors in CKL vs. SLD **(A)**, CKT *vs*. STD **(B)**, CKL *vs*. CKT **(C)**, and SLD *vs*. STD **(D)**.

As the main enrichment results are related to signal transduction pathways, and transcription factors often play an essential role in these pathways, we conducted further statistical analysis on the differentially expressed transcription factors. After freezing treatment, the differentially expressed transcription factors in Longyou 6 (CKL *vs*. SLD) and Tianyou 2 (CKT *vs*. STD) were similar, mainly MYB, bHLH, AP2-EREBP, NAC, and WRKY. However, the number of differentially expressed transcription factors in Tianyou 2 was significantly higher than that in Longyou 6, and the number of downregulated transcription factors in Tianyou 2 was generally significantly greater than the number of upregulated ones (Figures 5A,B). Interestingly, this difference was relatively small in Longyou 6, especially in some transcription factor families, such as MYB, bHLH, and WRKY, and the number of upregulated genes was significantly greater than that of downregulated genes (Figures 5A,B). In addition, before the freezing treatment, the number of differentially expressed transcription factors between the two varieties (CKL *vs*. CKT) was relatively low; however, the difference became particularly apparent after freezing treatment (SLD *vs*. STD), mainly reflected in MYB, bHLH, AP2-EREBP, NAC, and WRKY (Figures 5C,D). We also performed a Venn diagram analysis of the differentially expressed transcription factor families. There were 19 types of differentially expressed transcription factors in the two varieties, and these transcription factor family members could have played a pivotal role in the freezing response (Figure 5).

### Gene Expression Pattern Verification by qRT-PCR

**FIGURE 6:**
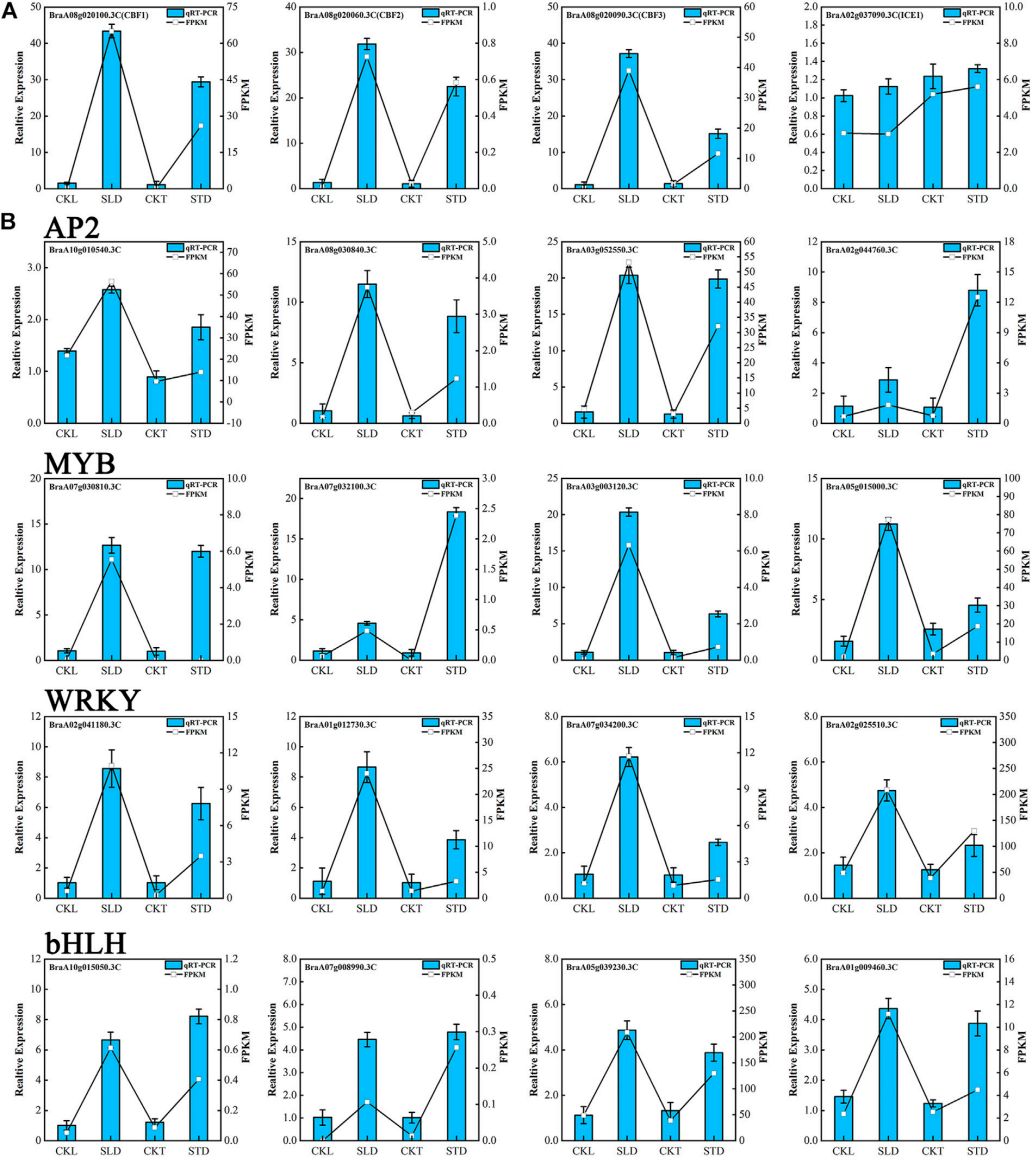
qRT-PCR analysis of cold response genes and transcription factors under freezing stress. **(A)** Expression of CBFs genes and ICE1. **(B)** Expression of cold response AP2, MYB, bHLH, and WRKY transcription factors. The relative expression level is indicated on the left *y*-axis. The FPKM from the RNA-Seq data are indicated on the right *y*-axis.

To validate the quality of the RNA-seq data, some genes and transcription factors associated with cold response or with a high fold-change in transcriptome data were selected for qRT-PCR analysis. CBFs are the core regulators of cold response signaling in plants (Shi et al., 2018; Ding et al., 2019). In *Arabidopsis,* six CBF/DREB1 genes have been characterized, *CBF1/DREB1C*, *CBF2/DREB1B*, and *CBF3/DREB1A* induced by cold, *CBF4/DREB1D*, *CBF5/DREB1E/DDF2*, and *CBF6/DREB1F/DDF1* induced by osmotic stresses, such as drought and salt stresses (Shinwari et al., 1998; Haake et al., 2002; Nakashima et al., 2009; Dietz et al., 2010). Our qRT-PCR data showed that *B. rapa CBF1*, *CBF2*, and *CBF3* were significantly induced by cold temperatures in Tianyou 2 and Longyou 6. The expression levels of *CBF1*, *CBF2*, and *CBF3* were higher in Longyou 6 than in Tianyou 2, and the qRT-PCR results also matched the FPKM data (Figure 6A). Unexpectedly, *B. rapa CBF4* and *CBF5* were also induced by freezing treatment in Tinayou 2 and Longyou 6, although the fold-change was low. In addition, some cold response genes, such as *RD29A*, *COR15B*, *KIN1*, *COR413*, and *KIN2*, were induced by freezing stress in Tinayou 2 and Longyou 6, and the fold changes of *RD29A*, *COR15B*, and *KIN1* were higher in Tinayou 2 than in Longyou 6 (Supplementary Figure S8). In contrast, ICE1, which is an important transcription regulator of the ICE1-CBF-COR cold signaling pathway, was only slightly induced by freezing stress, and the results are consistent with those of a previous study (Lee et al., 2005) (Figure 6A). We randomly selected twenty candidate genes from the AP2, bHLH, MYB, and WRKY transcription factor families, in which the changes were high before and after freezing treatment. qRT-PCR results showed that these candidate transcription factors can be obviously induced by freezing treatment (Figure 6B and Supplementary Figure S8). Although the fold-change in the expression level detected by qRT-PCR did not completely match the sequencing data, the expression patterns of all candidate genes under these two detection methods were similar. We confirmed that our sequencing data were reliable through mutual verification of the two methods.

### MAPK Cascades are Activated After Freezing Treatment

**FIGURE 7:**
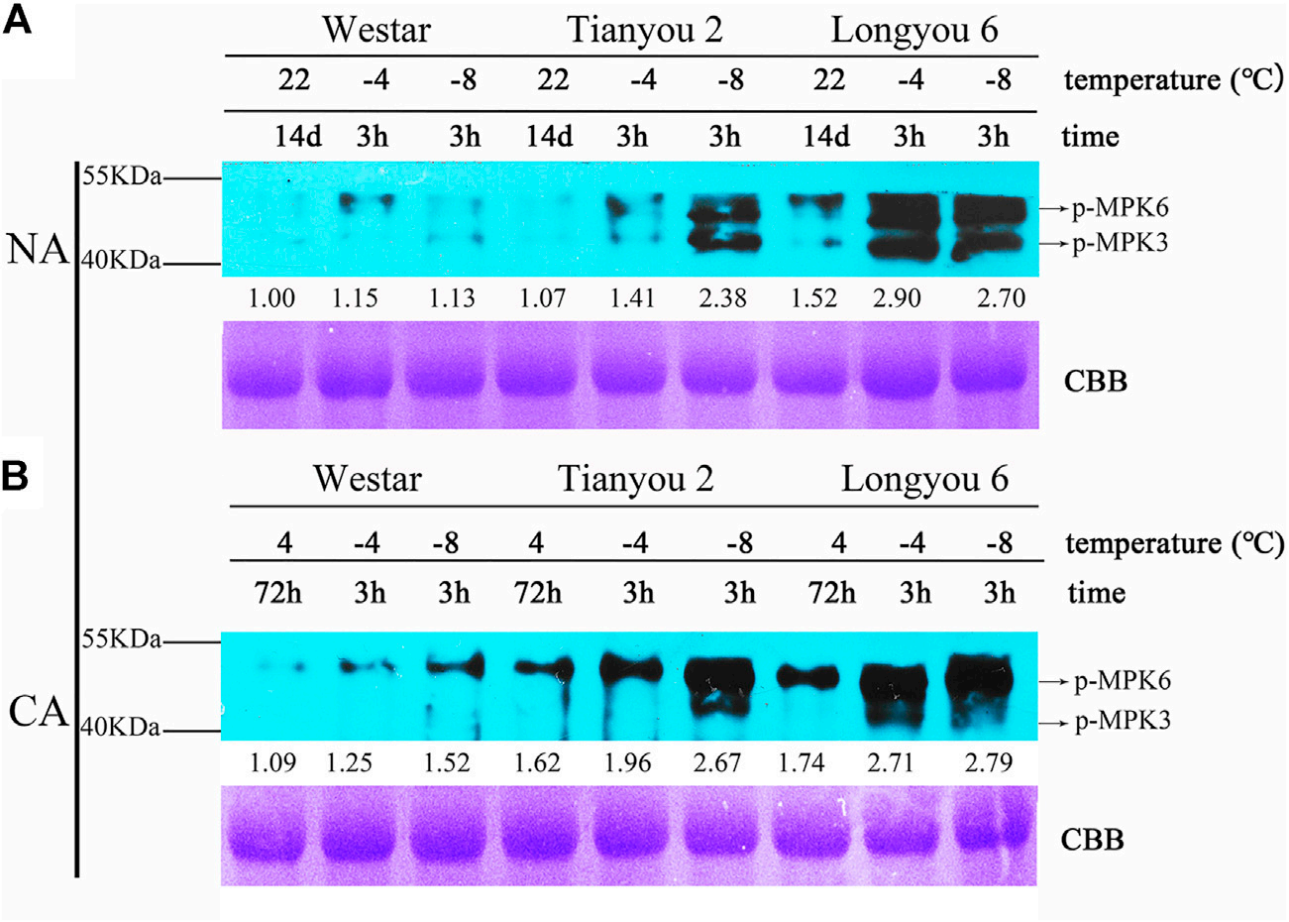
MPK3/6 Are Activated by freezing Treatments. 14-day-old NA **(A)** and CA **(B)** seedlings were treated at -4 and -8°C for 3 h. Total proteins were extracted and immunoblotting assays were performed using anti-pTEpY. The gel stained with Coomassie brilliant blue was used as a loading control.

KEGG pathway enrichment analysis of DEGs showed that the “MAPK signaling pathway-plant” was the most abundant of the four pathways after freezing treatment (SLD vs. STD). In order to corroborate whether freezing treatment can activate the MAPK cascades in *B. rapa*, immunoblotting was performed using an antibody against anti-pTEpY (phospho-p42/p44), which can recognize the phosphorylated/active form of MPK3, MPK4, and MPK6 (Jia et al., 2016). Immunoblotting results showed that the MPK6/3 phosphorylation levels of Longyou 6 and Tianyou 2 were rapidly activated by freezing treatment (−4°C/3 h and −8°C/3 h) with cold-acclimated (CA) and without cold-acclimated (NA) (Figures 7A,B), and the MAPK6/3 phosphorylation levels of Longyou 6 showed a higher increase than those of Tianyou 2 and Westar, even without cold treatment. After freezing treatment at −4°C for 3 h, the phosphorylation levels of Longyou 6 exhibited an increase of 2.9-fold, but those of Tianyou 2 and Westar showed an increase of only 1.41-fold and 1.15-fold, respectively (Figure 7A). Furthermore, the phosphorylation levels of Westar were only slightly activated by freezing treatment under NA treatment. These results indicated that freezing treatment could activate the MAPK signal cascades to regulate the freezing tolerance in *B. rapa*, particularly in Longyou 6.

## Discussion

**FIGURE 8:**
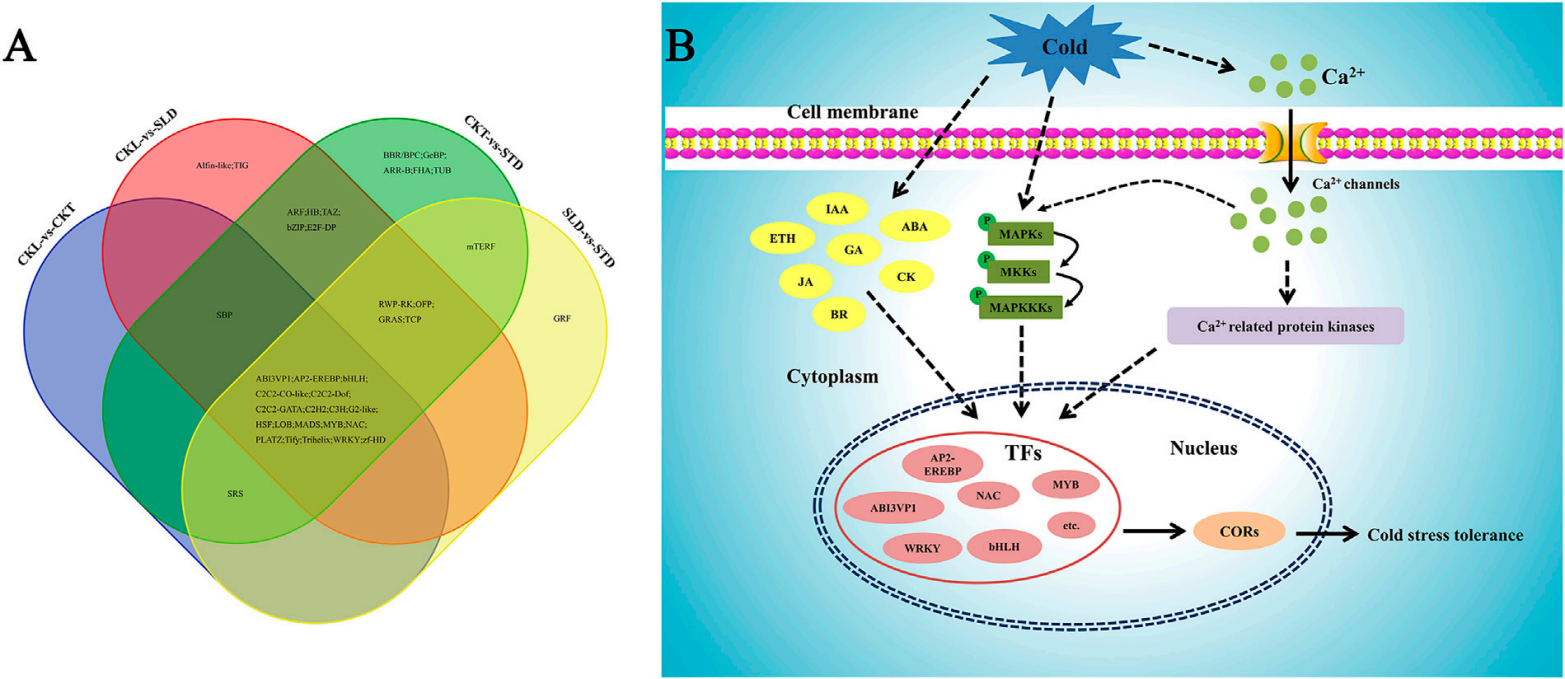
Venn diagram and speculative the pathway of *B. rapa* in response to freezing stress. **(A)** Venn diagram of differentially expressed transcription factors in different combinations before and after freezing treatment. **(B)** A proposed model of *B. rapa* in response to freezing stress. Under cold stress, the cell membrane of *B. rapa* first senses external cold signals, then the signals are transmitted to the nucleus by the MAPK cascades pathway, and many related transcription factors were induced to positively regulate plant freezing tolerance. Meanwhile, changes in hormone levels and Ca^2+^ concentration regulate related transcription factors to protect plants from freezing damage.

Low temperatures are a common environmental stress in plants, and multiple pathways are involved in the response of plants to low temperatures (Ding et al., 2019). Among them, changes in gene expression at the transcription level are an important part of plant response to cold stress. In *Arabidopsis,* approximately 3.9% of genes are regulated by cold stress (Lee et al., 2005). Therefore, studying the changes in gene expression at the transcriptional level is an effective strategy for understanding the molecular mechanisms of how plants respond to cold stress. Consistent with these studies, several researchers have also utilized transcriptome experiments to analyze gene expression changes in many crop species, including rice (Shen et al., 2014; Dasgupta et al., 2020), maize (Frey et al., 2020), wheat (Li et al., 2018), barley (Yuan et al., 2017), and tomato (Zhou et al., 2019). In this study, we used RNA sequencing technology to analyze and compare the transcriptome data of Longyou 6 (strong cold tolerance) and Tianyou 2 (weak cold tolerance) before and after freezing treatment and identified genes that may be involved in the cold tolerance pathway (Figure 8). These results provide a better understanding of the molecular mechanism of Longyou 6 in response to freezing stress and will facilitate the breeding of cold-tolerant *B. rapa* varieties.

The response of plants to cold stress can be divided into three stages. The first stage is the perception of cold stress by a different cold sensor, the second is the transduction of the low-temperature signal to the nucleus, and the third is the regulation of related downstream gene response to cold signals. Currently, the generally accepted view is that a change in cell membrane fluidity initiates low-temperature signals. This theory suggests that low temperatures can decrease the fluidity of cell membranes, and the hardening of cell membranes is accompanied by the rearrangement of the cytoskeleton, which stimulates the response of plants to low temperatures (Chen and Thelen, 2013). According to the results of the transcriptome data of Longyou 6 and Tianyou 2, regardless of rapeseed variety, after freezing stress, the GO classification results of their DEGs were similar (Figure 3), mainly involving “cellular process,” “membrane,” “membrane part,” and “binding.” This means that the two varieties have similar perception processes of freezing stress, conforming to the above theory. However, further GO enrichment analysis revealed that the DEGs in the two varieties have apparent differences in function (Supplementary Figure S6). After freezing treatment, the DEGs in Tianyou 2 (CKT *vs*. STD) were mainly involved in the “biosynthesis of substances”. In contrast, the DEGs in Longyou 6 (CKL *vs*. SLD) were primarily related to the “stimulation response” and “oxidoreductase activity”. Relevant studies have shown that oxidoreductase can protect rice chloroplasts from low-temperature damage (Sun et al., 2017), which implies that these DEGs are beneficial for improving the tolerance of Longyou 6 to cold damage. In addition, analysis of transcriptome data from the two varieties before freezing treatment (CKL vs. CKT) revealed that DEGs were mainly related to oxidoreductase, indicating that these DEGs were induced by the difference between the two varieties, highlighting the importance of these DEGs to the cold tolerance of *B. rapa*. However, after freezing treatment, the DEGs between the two varieties (SLD vs. STD) were mainly related to the “membrane composition”, suggesting that the two varieties may have significant differences in the transmission of cold signals.

The changes in DEGs before and after freezing treatment in the two varieties are consistent with the results of several previous studies. A study of rice showed that Nipponbare, a variety with a high chilling tolerance, accumulated ROS early and more rapidly than the low-chilling-tolerance 93–11 variety under chilling stress, revealing a ROS-dominated adaptation mechanism to chilling stress in the Nipponbare variety (Zhang et al., 2016). Consistent with the results of Zhang et al., 2016, our results of DEG and GO enrichment analyses also suggested that Longyou 6 has a higher ROS accumulation and more rapid ROS response than Tianyou 2 before and after freezing. Moreover, in *B. napus*, cold activation of BN115 (an ortholog of the *Arabidopsis COR15* gene) requires membrane rigidification (Sangwan et al., 2001). This suggests that membrane rigidification probably is a critical event for freezing tolerance in *B. napus*. Our analysis showed that DEGs between the two varieties (CKL vs. CKT) after freezing treatment (SLD vs. STD) were also mainly related to membrane composition, suggesting that membrane rigidification may be critical for the response of *Brassica* crops to freezing stress.

The response of plants to cold stress is a highly complex process involving the synergy of multiple pathways, during which numerous related proteins are translated so that the entire response process can run smoothly. Therefore, we classified DEGs by the KEGG pathways and found that in the two varieties, “signal transduction,” “carbohydrate metabolism,” and “translation” were the main biological processes (Supplementary Figures S5, S7). Among them, the importance of signal transduction in response to cold stress is indisputable, and involved throughout the response process. This signal transduction pathway may also play an important role in the cold tolerance of Longyou 6. Before freezing treatment, there was a substantial difference between the two varieties (CKL *vs*. CKT), mainly reflected in the MAPK signal transmission, hormone signal transduction, and carbohydrate metabolism pathways. After freezing treatment, these pathways were enriched in the two varieties (SLD *vs*. STD); the MAPK and hormone signal transduction pathways were more enriched than the carbohydrate metabolism pathway. Similarly, the pathways enriched in Longyou 6 (CKL *vs*. SLD) and Tianyou 2 (CKT *vs*. STD) before and after freezing treatment were roughly the same, suggesting that the carbohydrate metabolism pathway may affect the cold tolerance of Longyou 6. However, this effect was far less potent than the MAPK and hormone signaling pathways (Figure 4). Furthermore, our immunoblotting results showed that the phosphorylation level of MPK3/6 cascades could be rapidly activated by freezing treatment in *B. rapa*, and that Longyou 6 displayed a stronger basal MPK3/6 kinase activity than Tianyou 2 and Westar (Figure 7). The immunoblotting results were also consistent with the above-mentioned transcriptome analysis. In *Arabidopsis*, the cold-activated MPK3/6 phosphorylates the ICE1 protein to reduce its stability and negatively regulates CBF expression and freezing tolerance (Li et al., 2017; Zhao et al., 2017). However, in rice, the cold-activated OsMAPK3 phosphorylates and stabilizes OsICE1, consequently targeting OsTPP1 to increase trehalose levels and enhance chilling tolerance (Zhang et al., 2017). These reports suggest that cold signals regulated by MAPK cascades might be different in different species.

The cold-tolerance traits of plants are the result of the joint action of multiple genes. Only when clusters of cold-related genes are jointly activated by transcription can the cold-tolerance ability of plants be improved. In this process, transcription factors play a critical regulatory role, which can directly or indirectly regulate the expression of a series of downstream related genes. Analysis of the transcriptome data of Longyou 6 (CKL *vs*. SLD) and Tianyou 2 (CKT *vs*. STD) before and after the freezing process showed that after freezing treatment, the number of upregulated genes of MYB and bHLH transcription factor families in Longyou 6 was significantly higher than the number of downregulated ones (Figure 8A). CBF is a core member of the cold stress signal transduction pathway in plants (Thomashow, 1999). Our qRT-PCR results also showed that *CBF1*, *CBF2* and *CBF3* were significantly induced by freezing temperatures in Tianyou 2 and Longyou 6, but the expression levels of *CBF1*, *CBF2* and *CBF3* were high in Longyou 6, suggesting that Longyou 6 may have a more ingenious CBF-dependent cold regulation mechanism than Tianyou 2. ICE1, an important transcription regulator of the ICE1-CBF-COR cold signaling pathway, was only slightly induced by cold stress in our qRT-PCR analysis (Figure 6 and Supplementary Figure S8), consistent with the results of a previous study (Chinnusamy et al., 2003). These results suggest that Longyou 6 and Tianyou 2 might respond to freezing stimulation by regulating different transcription factors.

In summary, using the results of our transcriptome data, we investigated the main pathways in the response of *B. rapa* to freezing stress. When *B. rapa* is subjected to external cold stress, cell membrane might first perceive external signals through membrane rigidification. This signal is then transmitted to the nucleus through the cold-activated MAPK cascades signal transduction pathway, lead to produce related osmotic regulating substance to response to freezing stress. Meanwhile, though the changes of hormone levels and Ca^2+^ concentration regulate related transcription factors to protect *B. rapa* from freezing damage (Figure 8B). Although we did not fully elucidate the freezing resistance mechanisms of *B. rapa*, our findings provide a reference for the *B. rapa* response mechanism to freezing stress, from membrane rigidification to the MAPK cascades transduction pathway. Future research should focus on the gaps in our study findings, investigating which proteins sense membrane rigidification, and how the MAPK cascades regulates downstream transcription factors in response to freezing stress in *B. rapa*.

## Conclusions

This study used comparative transcriptome analysis to reveal the freezing tolerance signaling events in Longyou 6 (strong cold tolerance) and Tianyou 2 (weak cold tolerance) under different freezing stress. Phenotypic and physiological results confirmed that Longyou 6 may have a higher basal freezing resistance than Tinayou 2, and cold-acclimation can strengthen the freezing resistance of Longyou 6, while freezing stress can rapidly enhance the kinase activity of the MAPK cascades. Our results provide new insights into the response mechanism of *B. rapa* to freezing stress, which primarily involves cell membrane rigidification to perceive the cold signal, which is then transmitted to the nucleus along the cold-activated MAPK cascades pathway.

## Data Availability

The datasets presented in this study can be found in online repositories. The names of the repository/repositories and accession number(s) can be found below: The raw transcriptome reads have been deposited into the NCBI Short Read Archive (SRA) under accession number PRJNA782399.
