# Supplementary figures and images for: Comparative Transcriptome Analysis Revealed the Freezing Tolerance Signaling Events in Winter Rapeseed (Brassica rapa L.)
Source: Front Genet. 2022 Apr 26;13:871825. doi: 10.3389/fgene.2022.871825 (PMC9086196; doi:10.3389/fgene.2022.871825)

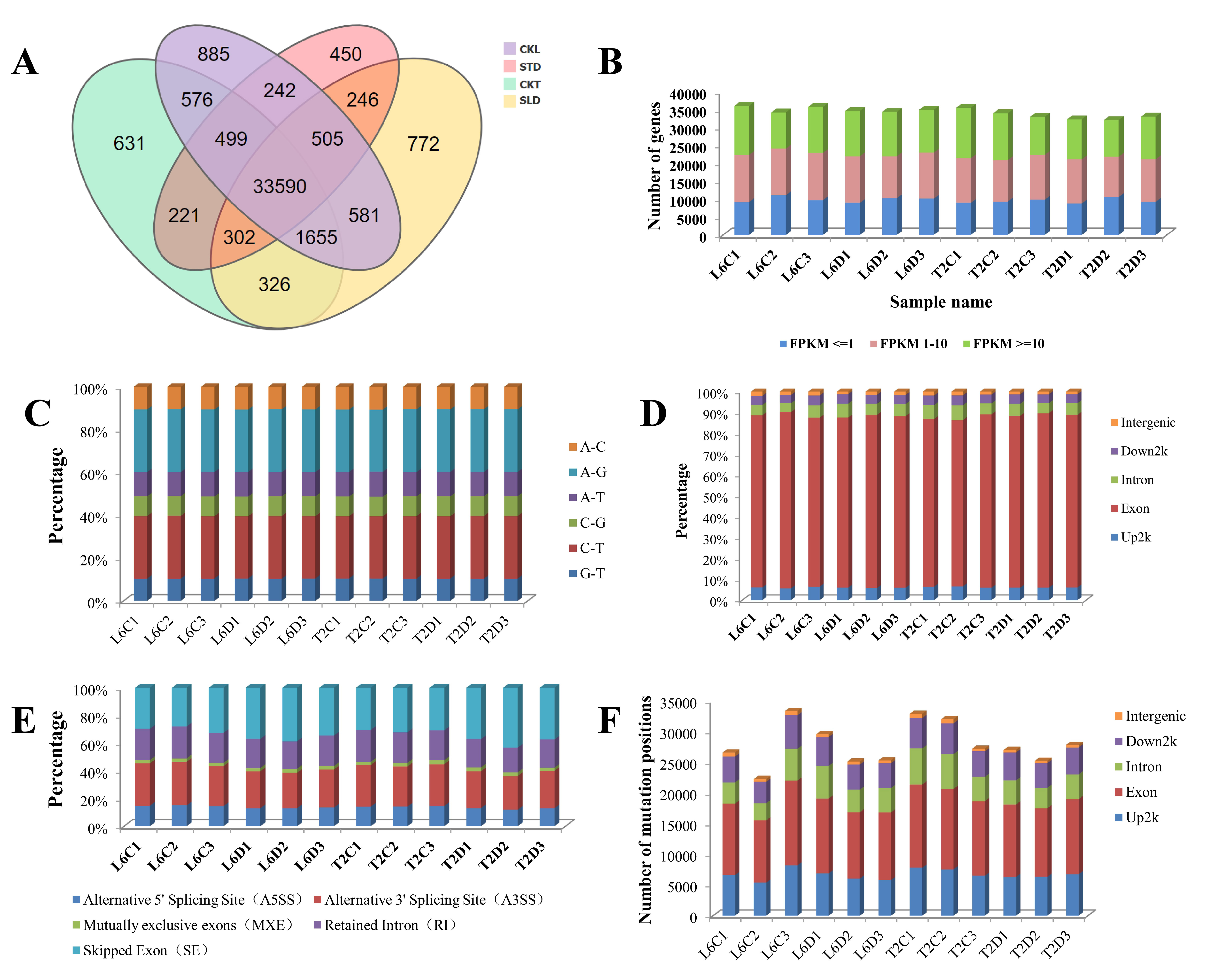

Supplement: Supplementary file 2 [file Image3.JPEG]

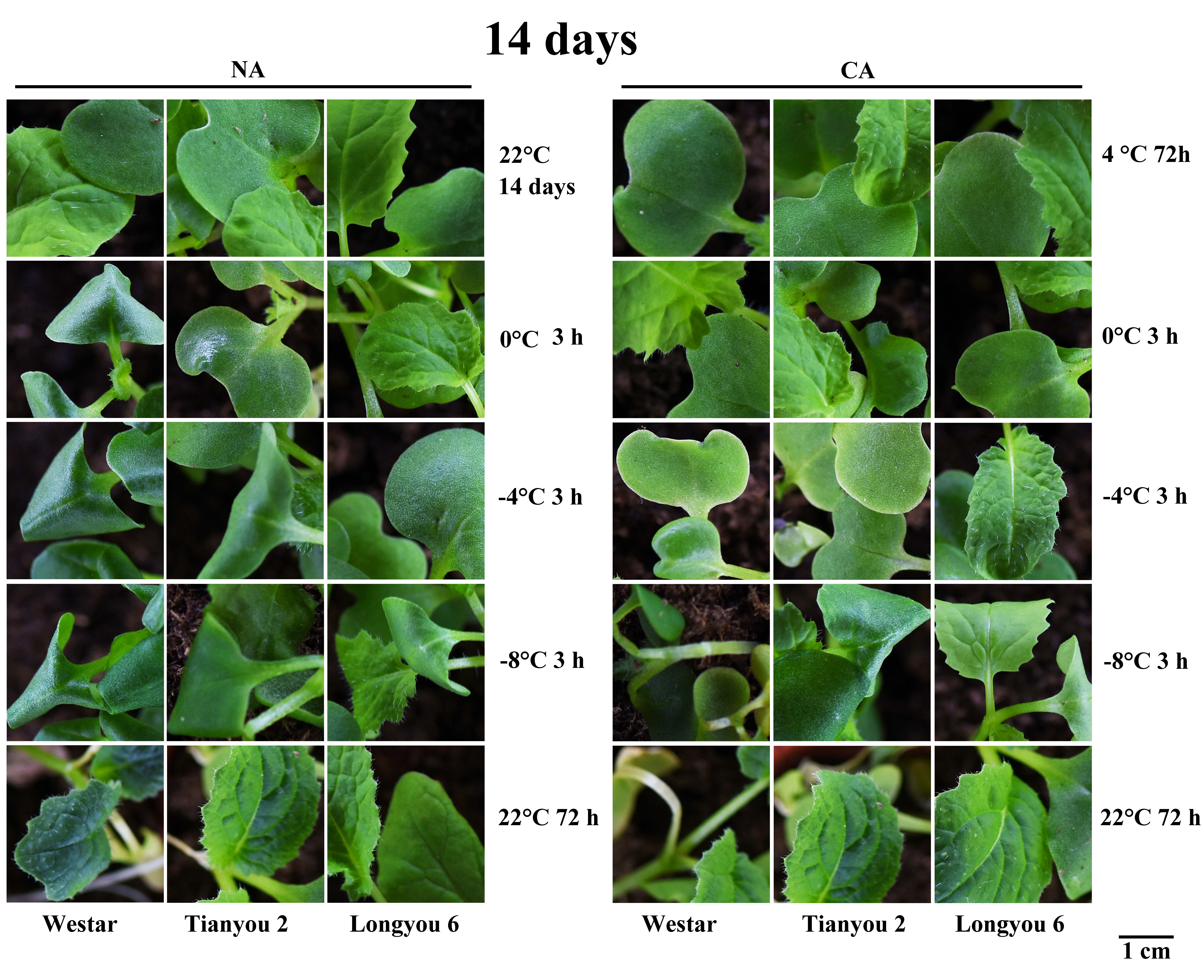

Supplement: Supplementary file 4 [file Image1.JPEG]

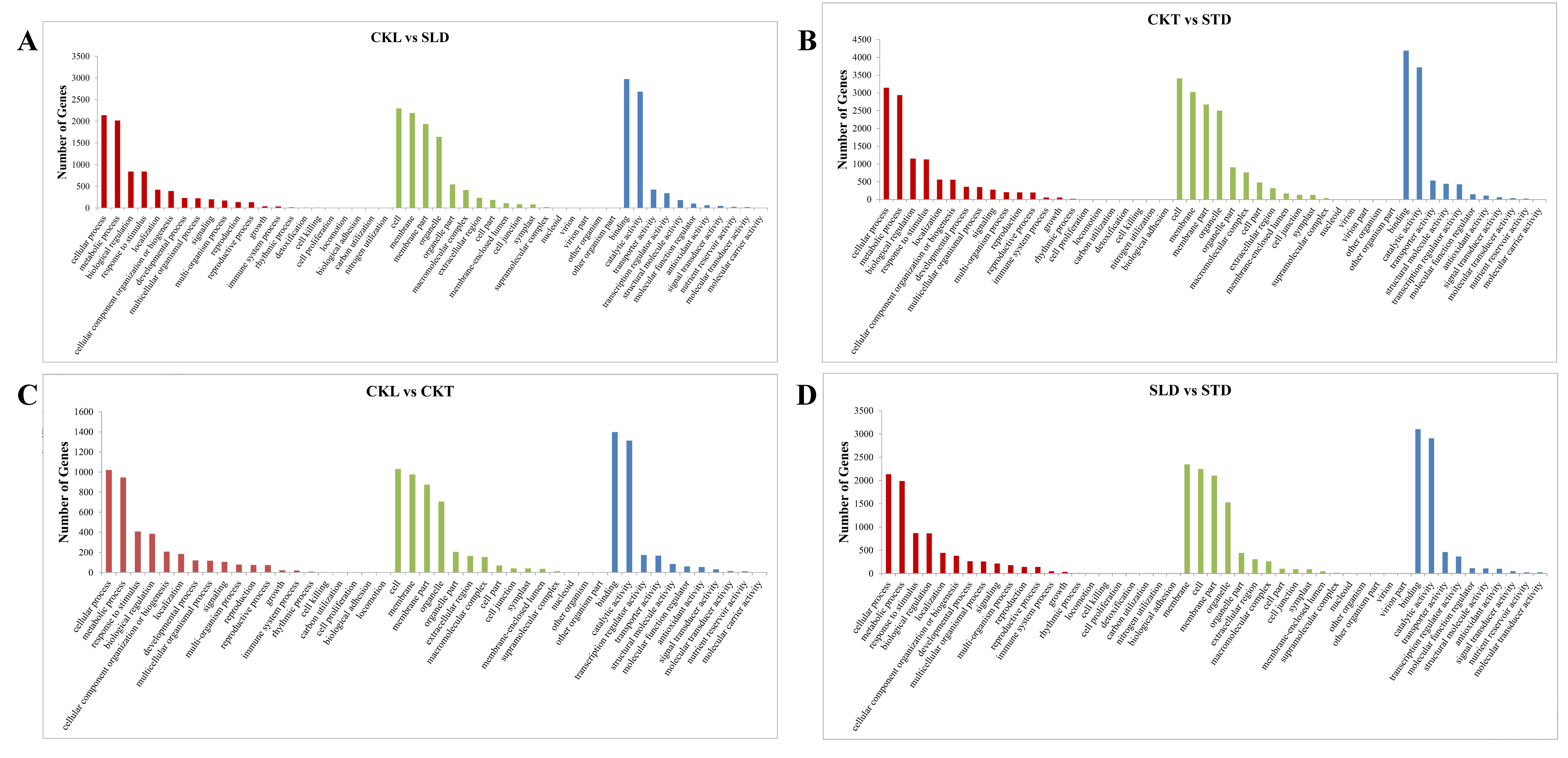

Supplement: Supplementary file 5 [file Image4.JPEG]

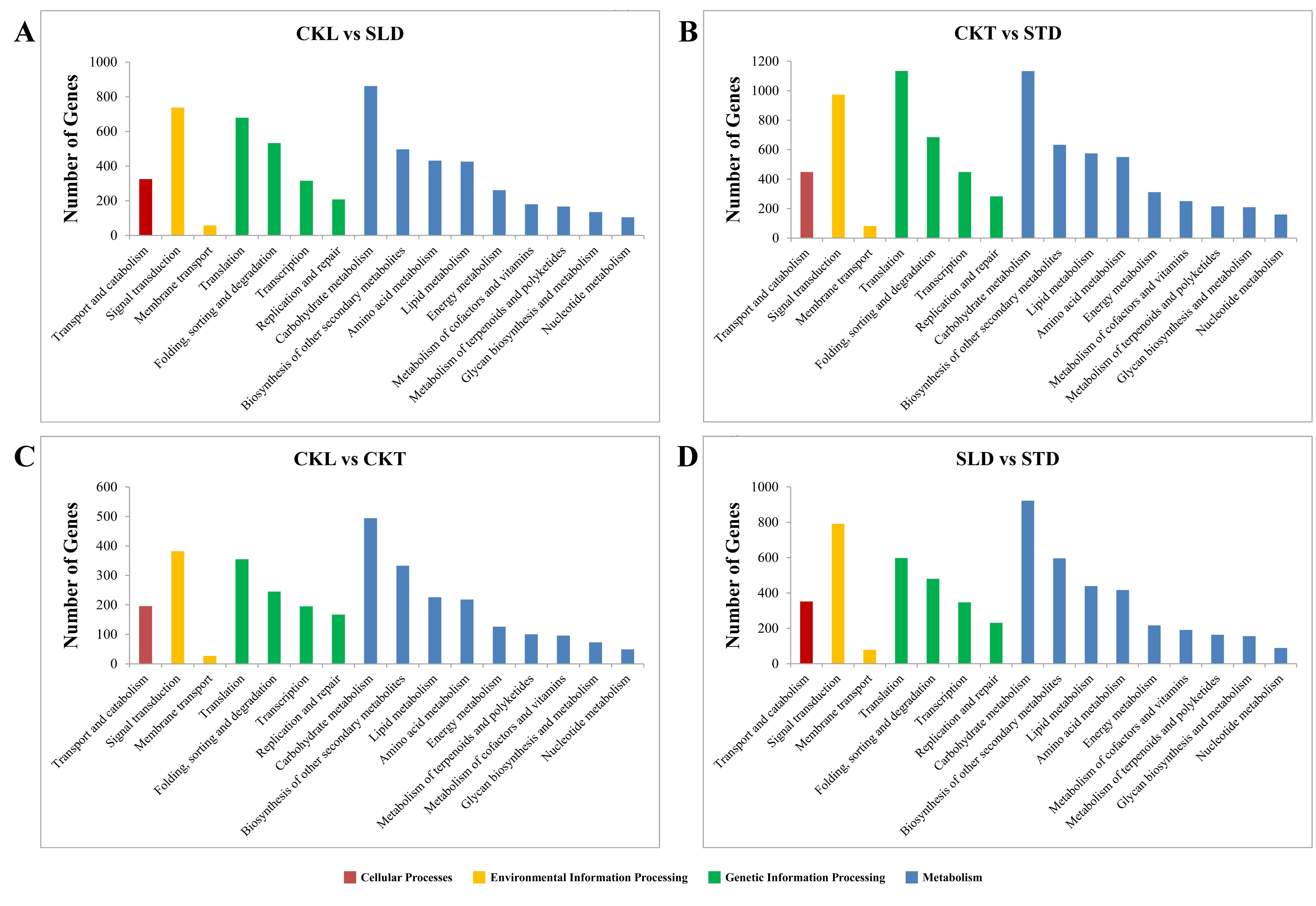

Supplement: Supplementary file 6 [file Image7.JPEG]

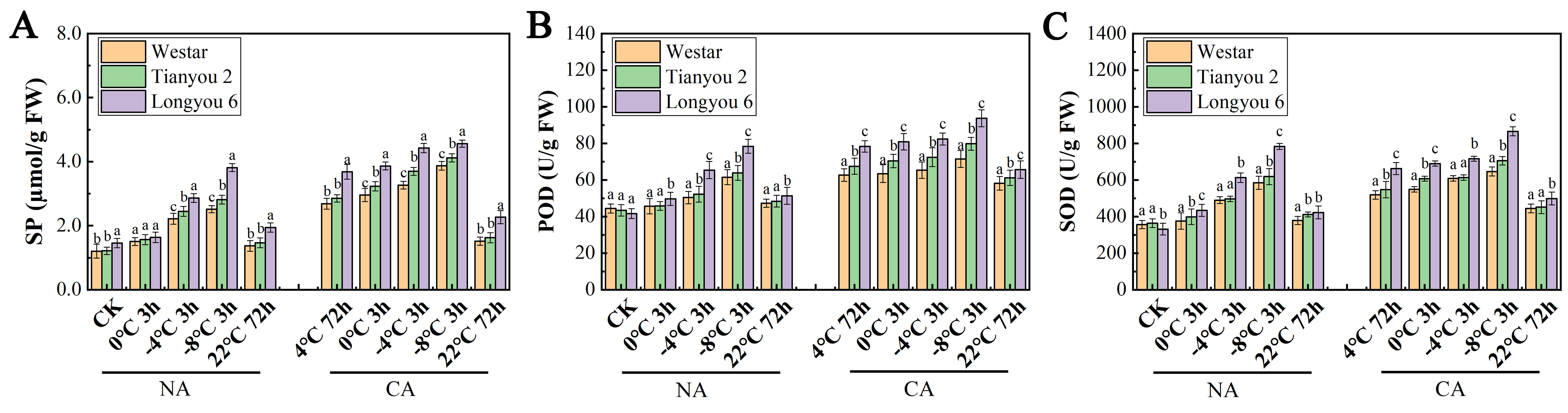

Supplement: Supplementary file 7 [file Image2.JPEG]

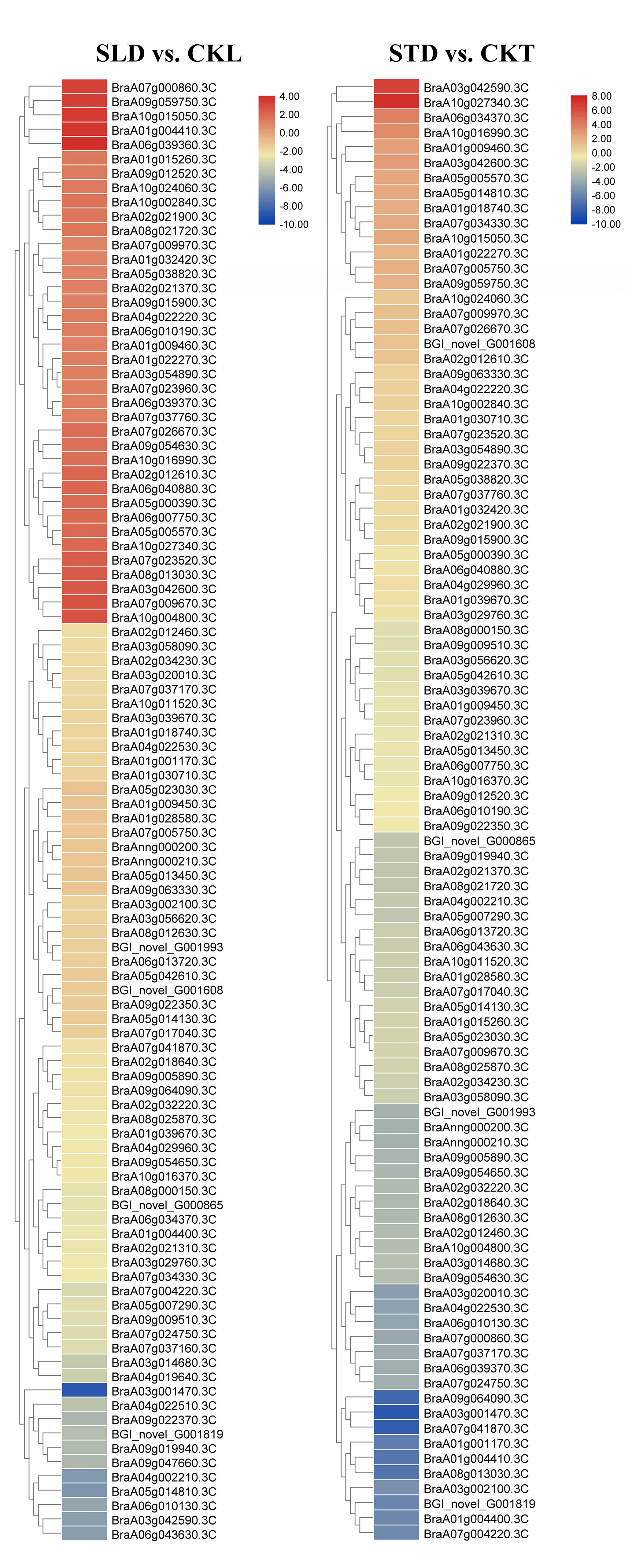

Supplement: Supplementary file 8 [file Image5.JPEG]

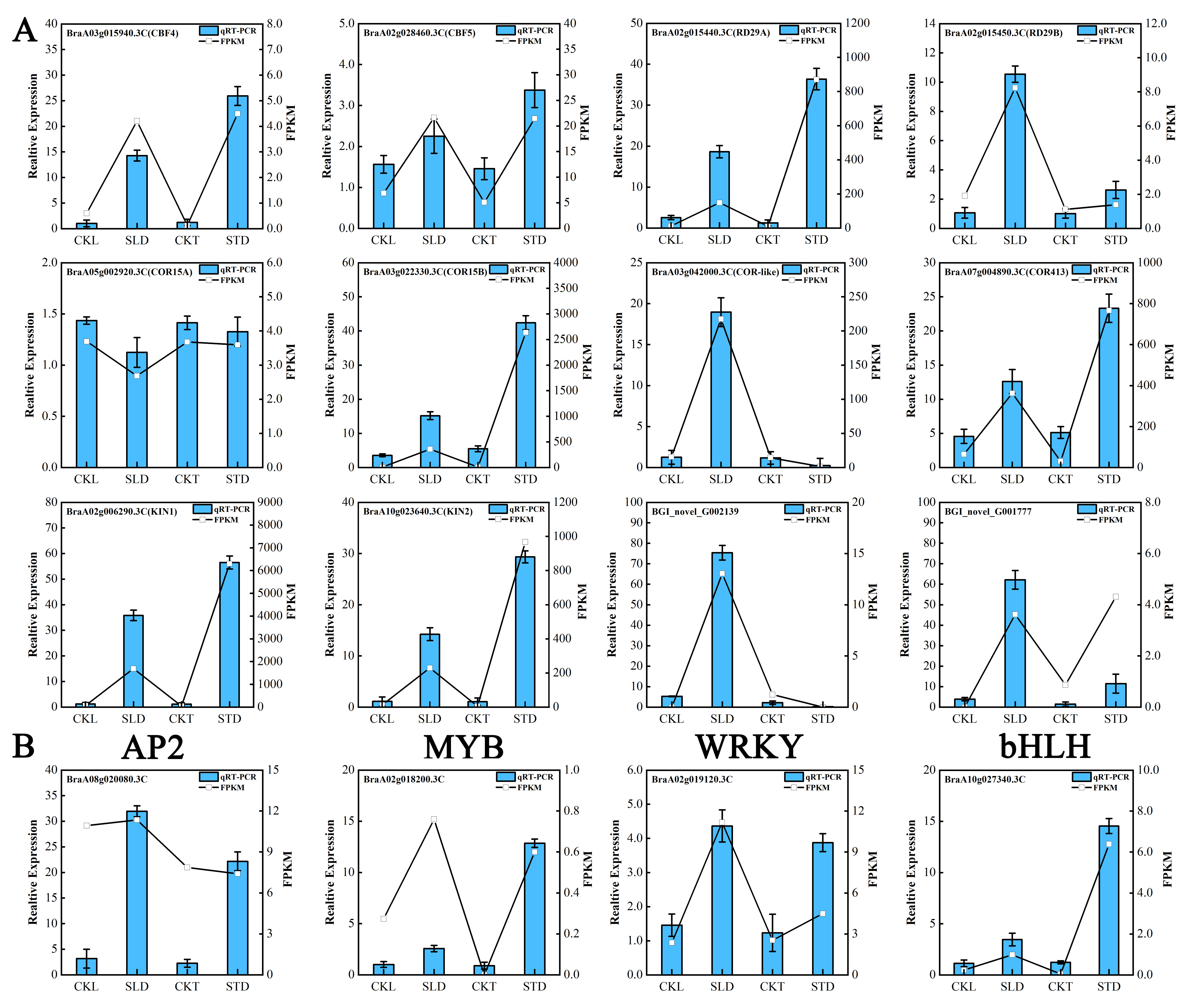

Supplement: Supplementary file 11 [file Image8.JPEG]

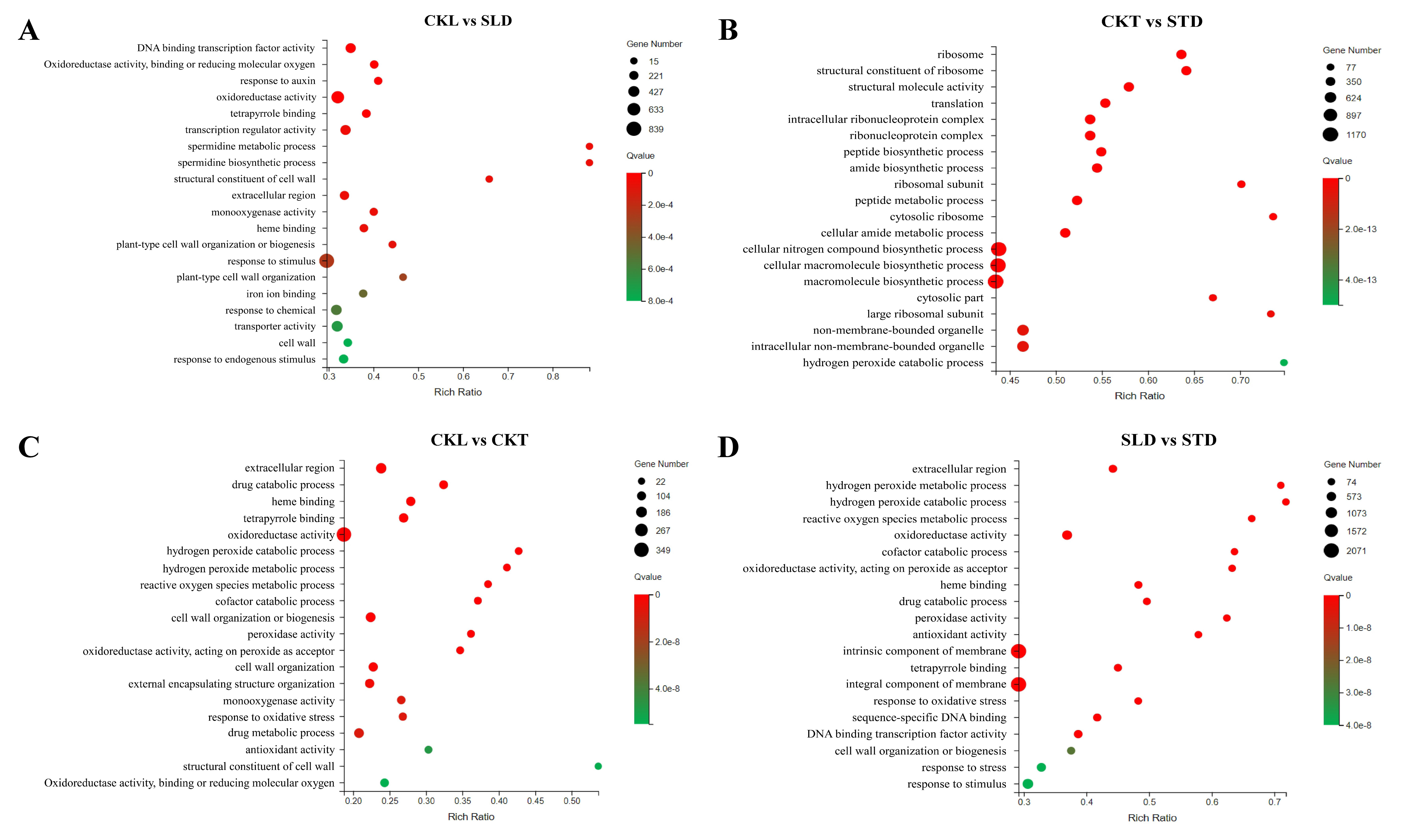

Supplement: Supplementary file 12 [file Image6.JPEG]
