# Supplementary material for: Comparative Transcriptome Analysis Revealed the Freezing Tolerance Signaling Events in Winter Rapeseed (Brassica rapa L.)
Source: Front Genet. 2022 Apr 26;13:871825. doi: 10.3389/fgene.2022.871825 (PMC9086196; doi:10.3389/fgene.2022.871825)
Supplement: Supplementary file 9 [file DataSheet1.PDF]

SUPPLEMENTARY MATERIAL FIGURE LEGENDS

FIGURE S1 | The symptom of enlarged leaves under freezing treatments.

FIGURE S2 | The physiochemical indicators of *B. rapa* under different freezing treatments.
(A) Soluble proteins (SP) contents. **(B)** Peroxidase (POD) activities. **(C)** Superoxide dismutase (SOD) activities.

FIGURE S3 | Differentially expressed genes types and distribution of SNPs and alternative splicing.
(A) Venn diagram of differentially expressed genes under freezing stress. **(B)** Genes number of different FPKM in different samples. **(C)** Types of SNPs in each sample. **(D)** Distribution of SNPs in each sample. **(E)** Types of alternative splicing in each sample. **(F)** Distribution of alternative splicing in each sample. T2C1 to T2C3 and T2D1 to T2D3 are three biological replicates of 14-day-old Tianyou 2 seedlings before and after freezing treatment, respectively. L6C1 to L6C3 and L6D1 to L6D1 are three biological replicates of 14-day-old Longyou 6 seedlings before and after freezing treatment, respectively.

FIGURE S4 | Functional classification of GO terms of DEGs under freezing stress.
The GO term can be further summarized into three functional categories: molecular functions, cellular components, and biological processes, which are distinguished by red, light green, and blue respectively in CKL vs. SLD **(A)**, CKT vs. STD **(B)**, CKL vs. CKT **(C)**, and SLD vs. STD **(D)**.

FIGURE S5 | Cluster analysis of DEGs of the key pathway under freezing stress.
(A) Upregulated GO and KEEG enrichment analysis in STD vs. CKT. **(B)** Downregulated GO and KEEG enrichment analysis in STD vs. CKT. **(C)** Upregulated GO and KEEG enrichment analysis in SLD vs. CKL. **(D)** Downregulated GO and KEEG enrichment analysis in SLD vs. CKL.

FIGURE S6 | The 20 most enriched GO terms among the DEGs identified in *B. rapa* under freezing treatments.
The most enriched GO terms in CKL vs. SLD **(A)**, CKT vs. STD **(B)**, CKL vs. CKT **(C)**, and SLD vs. STD **(D)**.

FIGURE S7 | KEGG pathway classification of DEGs in *B. rapa* under different freezing treatments.
KEGG pathway classification in CKL vs. SLD **(A)**, CKT vs. STD **(B)**, CKL vs. CKT **(C)**, and SLD vs. STD **(D)**. It is divided into 4 categories: Cellular Processes, Environmental Information Processing, Genetic Information Processing and

Metabolism, which are represented by red, yellow, green, and blue respectively.

FIGURE S8 | qRT-PCR analysis CBFs target genes and cold response transcription factors.
(A) Expression of CBF genes and their target genes. **(B)** Expression of cold response AP2, MYB, bHLH, and WRKY transcription factors.

TABLE S1| Overview of the RNA sequencing data.

TABLE S2| Differentially expressed genes of Longyou 6 and Tianyou 2 under freezing stress.

TABLE S3| Primer of qRT-PCR.
